# Prognostic value of small mother against decapentaplegic expression in human gastric cancer

**DOI:** 10.1080/21655979.2021.1935192

**Published:** 2021-06-17

**Authors:** He-Wei Zhang, Ying Guo, Lin-xiao Sun, Fu-biao Ni, Ke Xu

**Affiliations:** a Department of Surgery, Key Laboratory of Diagnosis and Treatment of Severe Hepato-Pancreatic Diseases of Zhejiang Province, Zhejiang Provincial Top Key Discipline in Surgery, The First Affiliated Hospital of Wenzhou Medical University, Wenzhou, Zhejiang, P.R. China; b Department of General Surgery, The Second Affiliated Hospital of Wenzhou Medical University, Wenzhou, Zhejiang, P.R. China; c Endocrinology Department, The First Affiliated Hospital of Wenzhou Medical University, Wenzhou, Zhejiang, P.R. China

**Keywords:** SMADs, gastric cancer, prognosis, Kaplan-Meier plotter

## Abstract

Gastric cancer is the fifth most common malignancy in the world with alow 5-year survival rate. To date, no study has investigated the prognostic role of the small mother against decapentaplegic (SMAD) in gastric cancer. The association of SMADs with overall survival (OS) of gastric cancer was analyzed on the online Kaplan-Meier (KM) plotter database. Clinical data such as stage, differentiation, gender, treatment, and Her2 mutation status of gastric cancer patients were analyzed. The (E)-SIS3 was used to inhibit SMAD3 expression in gastric cancer cells, and the effects of SMAD3 on gastric cancer cells were analyzed via real-time cellular analysis (RTCA), flow cytometry, colony formation, and immunofluorescence assay. The results showed that the high expression of three members of SMADs (SMAD1, SMAD2, SMAD4) was correlated with afavorable OS of gastric cancer patients. Meanwhile, SMAD3 expression level indicated highly differentiated cancer. We also observed that surgical treatment was associated with high expression level of SMAD1 and SMAD2. Besides, the effect of Her2 on gastric cancer was not noticeable. Moreover, (E)-SIS3 pharmacological assay revealed that inhibition of expression of SMAD3 suppressed the proliferation and migration ability of gastric cancer cells via inducing apoptosis. Collectively, these results demonstrate that the high expression level of three members of SMADs (SMAD1, SMAD2, and SMAD4) is significantly correlated with favorable OS of gastric cancer patients, which is opposite to SMAD3. Thus, SMADs regulate the differentiation of cancer and can be used to guide treatment decisions.

## Graphical abstract

**Figure:**
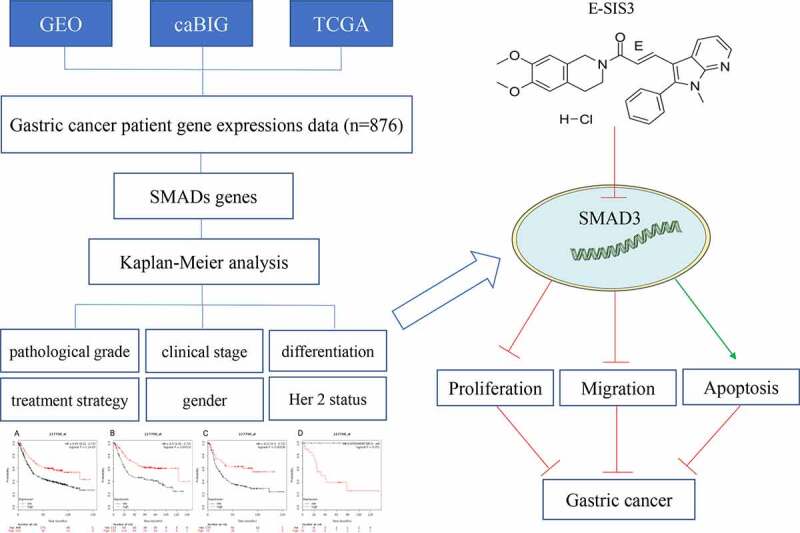

## Introduction

Gastric cancer is a malignant tumor of the gastric mucosal epithelium. It is the fifth most common malignant tumor and the second leading cause of cancer-related mortality worldwide. In addition, gastric cancer is the most common cancer in Eastern Asia [1,2]. Gastric cancer affects all regions of the stomach. The most common subtype of gastric cancer is adenocarcinoma. National Comprehensive Cancer Network (NCCN) guidelines recommend surgery, chemotherapy, and radiation therapy as effective treatments for gastric cancer. The primary treatment approach for resectable cancer is surgery through lymph node dissection [3]. The 5-year survival rate after surgery of early-stage gastric cancer patients is approximately 90%[4]. However, most gastric cancer patients are diagnosed at late stages and their 5-year survival rate is approximately 10% to 30% even after surgery [5]. Therefore, new prognostic markers of gastric cancer should be explored to improve the prognosis and quality of life of patients. Currently, three gastric cancer screening techniques are commonly used, including upper gastrointestinal endoscopy, serological testing, and ‘screen and treat’[6]. This study mainly focused on serological testing.

The transforming growth factor-β (TGF-β) family participates in the growth, differentiation, and apoptosis of cells. In addition, TGF-β has been implicated in embryonic development processes [7]. Notably, TGF-β is associated with the pathogenesis of several cancer types. In early-stage cancers, it restricts hyperplasia of epithelial cells and cancer cells. Furthermore, it induces cancer progression and metastasis in late-stage cancers [8]. SMADs are proteins mediated by two transmembrane receptors including TGF-β receptor type I and II heteromeric complex. A previous study reported that SMAD is a downstream intracellular protein in TGF-β signaling pathway [8]. Mammalian cells express nine different SMADs (SMAD 1–9), which are grouped into three types. The first type comprises SMADs 1, 2, 3, 5, 8 and 9 which are receptor-regulated or regulatory SMADs (R-SMADs). SMAD4 belongs to the second type, and it is a common SMAD (Co-SMAD). SMAD6 and SMAD7 are inhibitory or anti-SMADs (I-SMADs) and belong to type 3. Although a few differences exist among different types of SMADs which affect their respective functions, most of SMADs play related roles [7]. Currently, SMAD is associated with several cancers [9,10]. However, the relationship between SMAD and gastric cancer and its prognostic role in gastric cancer is still unclear.

In this study, we hypothesized that SMADs may influence the OS of gastric cancer patients. To prove this hypothesis, we retrieved data from the KM plotter by evaluating various clinicopathological characteristics (such as pathological grade, clinical stage, and treatment strategy) to explore the relationship between SMADs and the prognosis of human gastric cancer patients.

## Materials and methods

### Survival analysis of SMAD members

Expression levels of SMADs were evaluated and compared with overall survival (OS) of gastric cancer patients using an online KM plotter (http://kmplot.com/analysis) database. KM plotter was used to identify and validate the role of SMADs in gastric cancer. Gastric cancer patient gene expression data were retrieved from Gene Expression Omnibus, Cancer Biomedical Informatics Grid, and The Cancer Genome Atlas cancer datasets. Data retrieved included clinical characteristics of patients such as stage, differentiation, gender, treatment and Her2 mutation status of gastric cancer patients. In addition, data on clinical outcomes including clinical stages, differentiation, gender, chemotherapeutic strategy, and Her2 status were retrieved. Kaplan-Meier survival plots were generated using eight SMAD members (SMAD1, SMAD2, SMAD3, SMAD4, SMAD5, SMAD6, SMAD7, SMAD9) using the database Hazard ratio (HR). In this study, 95% confidence intervals and long rank P were used.

### Cells and reagents

Human gastric cancer cell lines AGS were obtained from American Type Culture Collection (ATCC, Manassas, USA). In addition, human gastric cancer cell lines MGC803 were obtained from Cell Bank of the Chinese Academy of Sciences (Shanghai, China). Cells were cultured in Dulbecco’s modified Eagle’s medium-high glucose (4.5 g/L) media (Thermo Fisher Scientific, Waltham, USA, C11995500BT), supplemented with 10% fetal bovine serum (Thermo Fisher Scientific, Waltham, USA,10099141), and 1% Penicillin-Streptomycin (Thermo Fisher Scientific, Waltham, USA, 15140122). Cells were then maintained at 37°C in a humidified chamber under 5% CO_2_. Media were changed every 1–2 days.

(E)-SIS3 was purchased from MedChemExpress library (CAS No.: 521984–48-5, Formula: C₂₈H₂₈ClN₃O₃, purity≥95.0%, MedChemExpress, USA). (E)-SIS3 was dissolved in dimethyl sulfoxide (DMSO) to generate 10 mM stock solution. The concentration of 10μM was chosen as the intermediate concentration to inhibit SMAD3 according to previous studies [11,12]. Experiments comprised three groups. In group 1, cells were treated with 0.1% DMSO (control group), in group 2, cells treated with (E)-SIS3 10 μM, whereas in group 3 cells were treated with (E)-SIS3 20 μM.

### Real-time cellular analysis

Cells were seeded on E16-plate (ACEA Biosciences, San Diego, USA) at 5 × 10^3^ cells/well for evaluation of proliferation [13]. Cell growth index was automatically recorded using a label-free real-time cell analysis system (RTCA; Roche, Penzberg, Germany). Cell growth index at each time point after cell treatment was normalized.

### Colony formation assay

Gastric cancer cells were pretreated with 0, 10, and 20 μM (E)-SIS3 for 24 h. Cells were then transferred to 6 well plates (1000 cells per well) and incubated for 2 weeks. After incubation, cells were stained with crystal violet stain and cell clones were counted. All experiments were carried out in triplicates.

### Cell migration assay

Gastric cancer cells were seeded in 6-well plates with 2 × 10^5^ cells per well and incubated for 48 h. The cell monolayer was then scratched with a 200 μL pipette tip to create a narrow gap space, as previously reported [14]. Cells were then washed three times with 0.01 M PBS and a medium containing 10 and 20 μM (E)-SIS3 was added. Cells were allowed to migrate and images of the culture area were obtained using an inverted microscope at 0, 24 and 48 h. All experiments were carried out in triplicates.

### Immunofluorescence assay

Gastric cancer cells treated with (E)-SIS3 (0,10, and 20 µM) for 24 h. Cells were then fixed with 4% formaldehyde (Sigma-Aldrich, Missouri, USA) for 20 min and permeabilized in 0.5% Triton X-100 (Sigma-Aldrich, T9284) for 10 min, as previously described [15]. Fixed cells were washed with 1x PBS, blocked with 5% BSA in 1x PBS at room temperature for 1 hour, and incubated overnight with Ki67 antibody (1:400) at 4°C. After incubation with Ki67 antibody, cells were washed three times with 1x PBS and incubated with CoraLite488 conjugated fluorescent secondary antibody (Proteintech, Cat No.:SA00013-2, 1:400) in PBST containing 1% BSA at 37°C for 1 hour. Cells were then washed twice and nucleus staining was performed using 4,6-diamidino-2-phenylindole (DAPI; Sigma-Aldrich, Missouri, USA, 10236276001). After staining, cells were visualized under an inverted fluorescence microscope (Nikon Eclipse Ti, Nikon, Japan). All experiments were performed in triplicates.

### Analysis of apoptosis through Flow-cytometry

Cells were treated with 0, 10, and 20μΜ (E)-SIS3 in a 6-well plate (5 × 10^5^cells/mL and 2 mL/well) and then washed with 0.01 M PBS. Cell apoptosis detection was performed using Dead Cell Apoptosis Kit (Thermo Fisher Scientific, Carlsbad, CA, USA, V13242). After attaining 85% confluence, cells were harvested and re-suspended in binding buffer with a density of 5 × 10^5^cells/mL. Furthermore, cells were incubated with 5 μL of Annexin V-FITC and 5 μL of propidium iodide (PI) for 15 min at room temperature under dark conditions, as previously described [16]. Flow cytometry was then performed using a FACSCalibur flow cytometer (BD Biosciences, USA). Data were analyzed using FlowJo software (version 10.0.7). All experiments were performed in triplicate.

### Quantitative RT-PCR analysis

Total RNA was isolated from cells by TRIzol reagent (Invitrogen, Carlsbad, CA, USA, 15596018). High-Capacity cDNA Reverse Transcription Kit (Thermo Fisher Scientific, 4368814) was used to synthesize complementary DNA (cDNA) from total RNA. Real-time PCR was performed at ABI PRISM 7500FAST PCR Sequence Detection System (Thermo Fisher Scientific) using the following parameters: 95°C for 10 mins, 40 cycles at 95°C for 10 s, at 60°C for 20 s and at 72°C for 30 s, and the fold changes of target genes between the experiment group and the control group were calculated using the 2^−ΔΔCt^ method. The primer pairs used for RT‑qPCR were as follows:

SMAD3 F: 5′-CAGCCATGTCGTCCATC-3′, R: 5′-CTCGCACCATTTCTCCTC-3′.

GAPDH F: 5′- CCTTCCGTGTCCCCACT-3′, R: 5′-GCCTGCTTCACCACCTTC-3′.

### Statistical analysis

Data were obtained from triplicate independent experiments and were presented as mean ± standard deviation. P < 0.05 was considered statistically significant. Statistical analysis was performed using SPSS version 18.0 (IBM, Armonk, USA) and GraphPad Prism version 6.0 (GraphPad Software Inc., San Diego, USA). One-way ANOVA and Student–Newman Keuls tests (SNK) were used to compare the mean values of between groups.

## Results

The transforming growth factor-β (TGF-β) family plays an important role in cell growth, differentiation and apoptosis. This study aims to systematically evaluate the prognostic role of SMADs in gastric cancer. We analyzed the relationship between SMADs and the overall survival rate (OS) of gastric cancer on the online Kaplan-Meier (KM) plotter database. The clinical data such as staging, differentiation, gender, treatment, and Her2 mutation status of patients with gastric cancer were analyzed. Through in vitro experiments, we further confirmed the effect of SMAD3 on the proliferation, migration and apoptosis of gastric cancer cells AGS and MGC803.

### Prognostic role of SMAD members in gastric cancer patients

**Figure 1.**
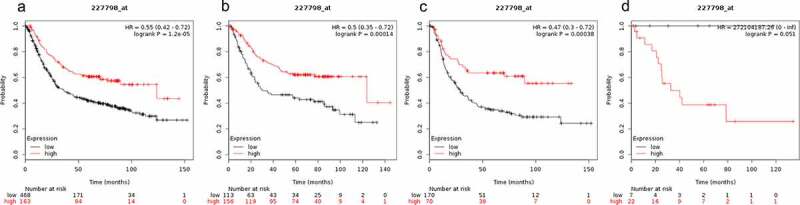
Prognostic value of SMAD1 expression in gastric cancer. Prognostic value of SMAD1 expression as shown by KM plotter (www.kmplot.com) curves. Affymetrix ID for SMAD1: 227798_ at. OS curves were plotted for A: all the patients (n = 876); B: intestinal cancer patients; C: diffuse cancer patients; D: mixed cancer patients

OS curves for gastrointestinal cancer patients, diffuse gastric cancer patients and mixed gastric cancer patients were plotted using KM plotter (www.kmplot.com). The prognostic value of SMAD1 mRNA expression in gastric cancer was explored. High expression levels of SMAD1 were highly correlated with favorable OS of gastric cancer patients (HR = 0.55 (0.42–0.72), P = 1.2e-05, Figure 1(a)), gastrointestinal cancer patients (HR = 0.5 (0.35–0.72), P = 0.00014, Figure 1(b)) and diffuse gastric cancer patients (HR = 0.47 (0.3–0.72), P = 0.00038 (Figure 1(c)). However, SMAD1 mRNA expression levels showed no correlation with OS of mixed gastric cancer patients (HR = 272104187.26 (0-inf), P = 0.051, Figure 1(d)).

**Figure 2.**
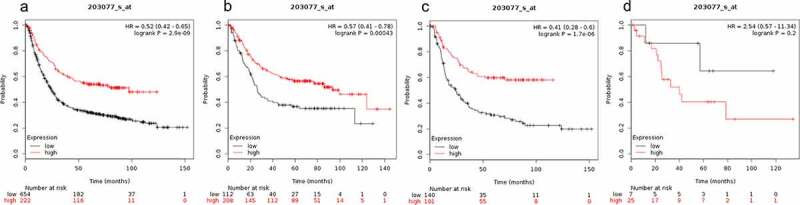
Prognostic value of SMAD2 expression in gastric cancer

The expression level of SMAD2 was not correlated with OS of mixed gastric cancer patients (HR = 2.54(0.57–11.34), P = 0.2 (Figure 2(d)). SMAD2 expression level was positively correlated with OS of gastrointestinal cancer patients (HR = 0.57 (0.41–0.78), P = 0.00043, Figure 2(b)) and diffuse gastric cancer patients (HR = 0.41 (0.28–0.6), P = 1.7e-0.6, Figure 2(c)). Therefore, SMAD2 is associated with better OS in gastrointestinal cancer and OS in diffuse gastric cancer patients (Figure 2(a)).

Prognostic value of SMAD2 expression as shown by KM plotter (www.kmplot.com) curves. Affymetrix ID for SMAD1: 203077_s_ at. OS curves were plotted for A: all the patients (n = 876); B: intestinal cancer patients; C: diffuse cancer patients; D: mixed cancer patients.

**Figure 3.**
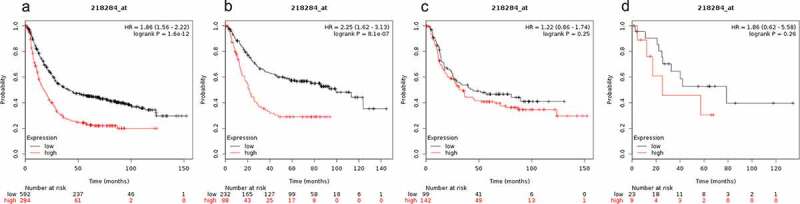
Prognostic value of SMAD3 expression in gastric cancer

High SMAD3 mRNA expression level was associated with a poor OS in all GC patients. In addition, SMAD3 mRNA expression level was not significantly correlated with OS of mixed gastric cancer patients. Results of the KM plotter analysis showed that SMAD3 mRNA expression level affected the OS of all GC patients (1.86 (1.56–2.22), P = 1.6e-12, Figure 3(a)) and OS of gastrointestinal cancer patients (2.25 (1.62–3.13), P = 8.1e-07, Figure 3(b)). On the contrary, SMAD3 mRNA expression levels showed no significant correlation with OS of diffuse gastric cancer patients (1.22 (0.86–1.74), P = 0.25, Figure 3(c)) and OS of mixed gastric cancer patients (HR = 1.86 (0.62–5.58), P = 0.26, Figure 3(d)).

Prognostic value of SMAD3 expression as shown by KM plotter (www.kmplot.com) curves. Affymetrix ID for SMAD3: 218284_ at. OS curves were plotted for A: all the patients (n = 876); B: intestinal cancer patients; C: diffuse cancer patients; D: mixed cancer patients.

**Figure 4.**
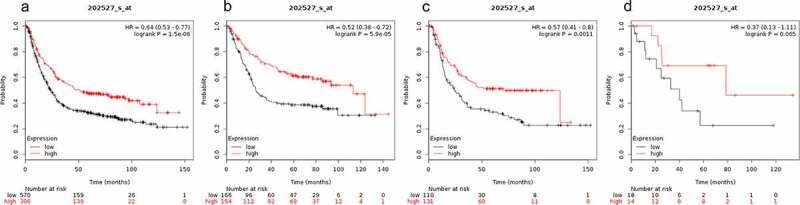
Prognostic value of SMAD4 expression in gastric cancer

SMAD4 expression was significantly correlated with better OS for all gastric cancer patients (HR = 0.64 (0.53–0.77) P = 1.5e-06, Figure 4(a)), OS of gastrointestinal cancer patients (HR = 0.52 (0.38–0.72) P = 5.9e-05, Figure 4(b)) and OS of diffuse gastric cancer patients (HR = 0.57 (0.41–0.8), P = 0.0011, Figure 4(c)). However, SMAD4 mRNA expression was not correlated with OS for the mixed gastric cancer patients (HR = 0.37 (0.13–1.11) P = 0.065, Figure 4(d)).

Prognostic value of SMAD4 expression as shown by KM plotter (www.kmplot.com) curves. Affymetrix ID for SMAD4: 202527 _ s _ at. OS curves were plotted for A: all the patients (n = 876); B: intestinal cancer patients; C: diffuse cancer patients; D: mixed cancer patients.

**Figure 5.**
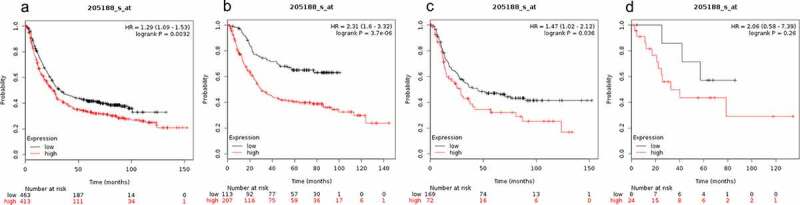
Prognostic value of SMAD5 expression in gastric cancer

The prognostic value of SMAD5 mRNA expression in gastric cancer is presented in Figure 5. Low SMAD5 mRNA expression level was significantly correlated with better OS in all gastric cancer patients (HR = 1.29 (1.09–1.52) P = 0.0032, Figure 5(a)) and better OS in diffuse gastric cancer patients (HR = 1.47 (1.02–2.12) P = 0.036 (Figure 5(c)). These findings show that low expression level of SMAD5 in gastrointestinal cancer results in higher OS in gastric cancer patients (HR = 2.31 (1.6–3.32) P = 3.7e-06, Figure 5(b)). However, over expression of SMAD5 was not correlated with OS in mixed gastric cancer patients (HR = 2.06 (0.58–7.39), P = 0.26, Figure 5(d)).

Prognostic value of SMAD5 expression as shown by KM plotter (www.kmplot.com) curves. Affymetrix ID for SMAD5: 205188 _ s _ at. OS curves were plotted for A: all the patients (n = 876); B: intestinal cancer patients; C: diffuse cancer patients; D: mixed cancer patients.

**Figure 6.**
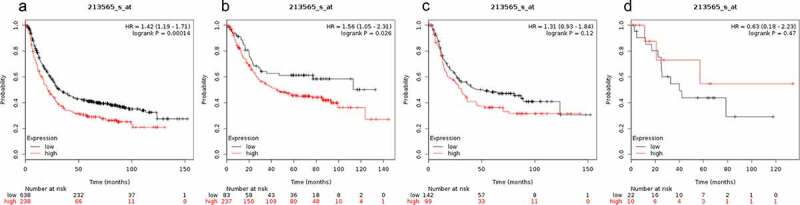
Prognostic value of SMAD6 expression in gastric cancer

Prognostic value of SMAD6 expression in gastric cancer patients is shown in Figure 6. Higher expression level of SMAD6 was not significantly correlated with OS for mixed gastric cancer (HR = 0.63 (0.18–2.23), P = 0.47, Figure 6(d)) and OS of diffuse gastric cancer patients (HR = 1.31 (0.93–1.84), P = 0.12, Figure 6(c)). However, expression level of SMAD6 was significantly correlated with OS of all gastric cancer patients (HR = 1.42 (1.19–1.71), P = 0.00014 (Figure 6(a)) and gastrointestinal cancer patients (HR = 1.56 (1.05–2.31), P = 0.026 (Figure 6(b)).

Prognostic value of SMAD6 expression as shown by KM plotter (www.kmplot.com) curves. Affymetrix ID for SMAD6: 213565 _ s _ at. OS curves were plotted for A: all the patients (n = 876); B: intestinal cancer patients; C: diffuse cancer patients; D: mixed cancer patients.

**Figure 7.**
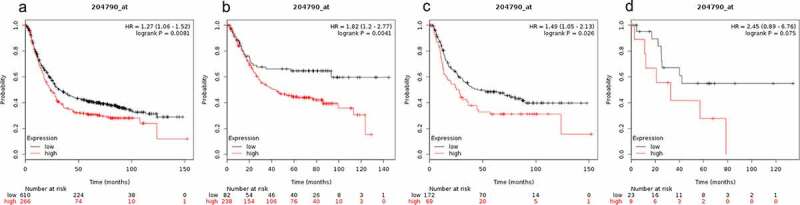
Prognostic value of SMAD7 expression in gastric cancer

Prognostic value of SMAD7 expression in gastric cancer patients is presented in Figure 7. SMAD7 expression level was not correlated with OS for mixed gastric cancer patients (HR = 2.45 (0.89–6.76), P = 0.075, Figure 7(d)). Low expression of SMAD7 was a favorable predictor of OS in all gastric cancer patients (HR = 1.27 (1.06–1.52), P = 0.0081, Figure 7(a)), gastrointestinal cancer patients (HR = 1.82 (1.2–2.77), P = 0.0041, Figure 7(b)), and diffuse gastric cancer patients (HR = 1.49 (1.05–2.13), P = 0.026, Figure 7(c)).

Prognostic value of SMAD7 expression as shown by KM plotter (www.kmplot.com) curves. Affymetrix ID for SMAD7: 204790 _ at. OS curves were plotted for A: all the patients (n = 876); B: intestinal cancer patients; C: diffuse cancer patients; D: mixed cancer patients.

**Figure 8.**
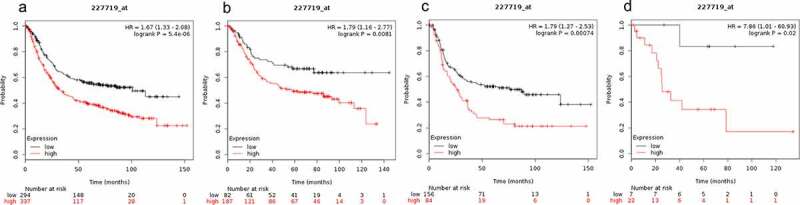
Prognostic value of SMAD9 expression in gastric cancer

Low expression level of SMAD9 was correlated with better OS in all gastric cancer patients (HR = 1.67 (1.33–2.08), P = 5.4e-06, Figure 8(a)), gastrointestinal cancer patients (HR = 1.79 (1.16–2.77), P = 0.0081, Figure 8(b)), diffuse gastric cancer patients (HR = 1.79 (1.27–2.53), P = 0.00074, Figure 8(c)), and mixed gastric cancer patients (HR = 7.86 (1.01–60.93), P = 0.02, Figure 8(d)).

Prognostic value of SMAD9 expression as shown by KM plotter (www.kmplot.com) curves. Affymetrix ID for SMAD9: 227719 _ at. OS curves were plotted for A: all the patients (n = 876); B: intestinal cancer patients; C: diffuse cancer patients; D: mixed cancer patient.

These findings show that high expression level of SMAD9 is correlated with high risk of all gastric cancer types including gastrointestinal cancer, diffuse gastric cancer, and mixed gastric cancer. On the contrary, high expression levels of SMAD1, SMAD2, and SMAD4 showed protective effects. To further explore the role of SMADs in gastric cancer, clinicopathological features such as pathological stages (Table 1), differentiation (Table 2), gender (Table 3), treatment strategies (Table 4) and Her2 status (Table 5) were evaluated.

**Table 1.** Correlation of SMAD gene expression level with overall survival (OS) of gastric cancer patients in different pathological stages

SMADs	Clinic stages	Cases	HR (95% CI)	P-value
SMAD1	1	62	0.18 (0.06 − 0.59)	0.0016*
	2	135	0.48 (0.23 − 1.01)	0.048*
	3	197	0.59 (0.4 − 0.86)	0.0053*
	4	140	0.8 (0.54 − 1.18)	0.25
SMAD2	1	67	0.22 (0.07 − 0.69)	0.0046*
	2	140	0.52 (0.23 − 1.17)	0.11
	3	305	0.4 (0.28 − 0.59)	1.6e−06*
	4	148	0.71 (0.47 − 1.09)	0.12
SMAD3	1	67	0.42 (0.15 − 1.15)	0.081
	2	140	2.24 (1.04 − 4.81)	0.035*
	3	305	1.72 (1.29 − 2.29)	0.00017*
	4	148	0.68 (0.46 − 1)	0.047*
SMAD4	1	67	0.34 (0.12 − 0.99)	0.039*
	2	140	0.71 (0.37 − 1.35)	0.29
	3	305	0.42 (0.3 − 0.6)	8e−07*
	4	148	0.83 (0.55 − 1.25)	0.37
SMAD5	1	67	5.43 (0.71 − 41.71)	0.068
	2	140	2.64 (1.44 − 4.84)	0.0011*
	3	305	1.74 (1.24 − 2.45)	0.0012*
	4	148	2 (1.31 − 3.05)	0.001*
SMAD6	1	67	1.66 (0.53 − 5.17)	0.38
	2	140	2.55 (1.18 − 5.49)	0.013*
	3	305	1.4 (1.05 − 1.86)	0.022*
	4	148	1.4 (0.95 − 2.05)	0.084
SMAD7	1	67	3.4 (1.25 − 9.25)	0.011*
	2	140	1.87 (1.02 − 3.44)	0.04*
	3	305	1.35 (1.01 − 1.8)	0.04*
	4	148	0.76 (0.51 − 1.14)	0.19
SMAD9	1	62	4.42 (0.97 − 20.11)	0.036*
	2	135	2.17 (1.03 − 4.56)	0.037*
	3	197	1.44 (0.97 − 2.15)	0.067
	4	140	1.61 (1.08 − 2.39)	0.019*

*P < 0.05

Abbreviations: CI, confidence interval; HR, hazard ratio.

**Table 2.** Correlation of SMAD gene expression level with overall survival (OS) of gastric cancer patients with different differentiation

SMADs	Differentiation	Cases	HR (95% CI)	P-value
SMAD1	Poor	121	1.51 (0.91 − 2.5)	0.11
	Moderate	67	0.5 (0.25 − 1.01)	0.048*
	Well	32	Not available	Not available
SMAD2	Poor	165	1.22 (0.81 − 1.85)	0.34
	Moderate	67	0.59 (0.29 − 1.18)	0.13
	Well	32	0.6 (0.25 − 1.42)	0.24
SMAD3	Poor	165	1.26 (0.82 − 1.95)	0.29
	Moderate	67	2.45 (1.22 − 4.93)	0.0099*
	Well	32	3.97 (0.92 − 17.13)	0.047*
SMAD4	Poor	165	0.81 (0.53 − 1.24)	0.34
	Moderate	67	0.5 (0.25 − 1.02)	0.054
	Well	32	0.35 (0.14 − 0.89)	0.021*
SMAD5	Poor	165	0.81 (0.55 − 1.21)	0.31
	Moderate	67	1.74 (0.8 − 3.82)	0.16
	Well	32	0.47 (0.19 − 1.15)	0.09
SMAD6	Poor	165	1.64 (1.1 − 2.44)	0.014*
	Moderate	67	0.6 (0.28 − 1.28)	0.18
	Well	32	0.55 (0.23 − 1.31)	0.17
SMAD7	Poor	165	1.62 (1.08 − 2.45)	0.019*
	Moderate	67	2.08 (0.85 − 5.13)	0.1
	Well	32	2.72 (0.8 − 9.27)	0.097
SMAD9	Poor	121	1.42 (0.82 − 2.43)	0.21
	Moderate	67	1.81 (0.79 − 4.14)	0.15
	Well	32	Not available	Not available

*P < 0.05

Abbreviations: CI, confidence interval; HR, hazard ratio.

**Table 3.** Correlation of SMAD gene expression level with overall survival (OS) of gastric cancer patients of different genders

SMADs	Gender	Cases	HR (95% CI)	P-value
SMAD1	Female	187	0.39 (0.21 − 0.7)	0.0011*
	Male	349	0.65 (0.48 − 0.87)	0.0038*
SMAD2	Female	236	0.53 (0.36 − 0.8)	0.0021*
	Male	545	0.48 (0.36 − 0.62)	3.9e−08*
SMAD3	Female	236	2.26 (1.57 − 3.25)	6.2e−06*
	Male	545	1.83 (1.47 − 2.27)	3.3e−08*
SMAD4	Female	236	0.62(0.44–0.88)	0.0075*
	Male	545	0.55 (0.43 − 0.71)	1.4e−06*
SMAD5	Female	236	1.27 (0.9 − 1.81)	0.17
	Male	415	1.41 (1.14 − 1.75)	0.0015*
SMAD6	Female	236	1.73 (1.21 − 2.48)	0.0023*
	Male	545	1.38 (1.1 − 1.73)	0.0053*
SMAD7	Female	236	1.64 (1.15 − 2.35)	0.0059*
	Male	495	1.17 (0.93 − 1.48)	0.17
SMAD9	Female	187	2.54 (1.38 − 4.67)	0.002*
	Male	349	1.66 (1.23 − 2.22)	0.00068*

*P < 0.05

Abbreviations: CI, confidence interval; HR, hazard ratio.

**Table 4.** Correlation of SMAD gene expression level with overall survival (OS) of gastric cancer patients under different treatment strategies

SMADs	Treatments	Cases	HR (95% CI)	P-value
SMAD1	Surgery alone	380	0.69 (0.49 − 0.97)	0.03*
	5-FU-based adjuvant	34	0.52 (0.21 − 1.31)	0.16
	Other adjuvant	76	0.5 (0.2 − 1.25)	0.13
SMAD2	Surgery alone	380	0.65 (0.48 − 0.89)	0.0068*
	5-FU-based adjuvant	153	1.31 (0.89 − 1.93)	0.16
	Other adjuvant	76	0.56 (0.23 − 1.38)	0.2
SMAD3	Surgery alone	380	1.41 (1 − 1.99)	0.051
	5-FU-based adjuvant	153	2.23 (1.46 − 3.42)	0.00015*
	Other adjuvant	76	1.85 (0.67 − 5.1)	0.23
SMAD4	Surgery alone	380	0.75 (0.56 − 1)	0.046*
	5-FU-based adjuvant	153	0.72 (0.49 − 1.04)	0.077
	Other adjuvant	76	0.45 (0.13 − 1.52)	0.18
SMAD5	Surgery alone	380	1.36 (0.97 − 1.91)	0.07
	5-FU-based adjuvant	153	0.38 (0.25 − 0.57)	1.3e−06*
	Other adjuvant	76	0.4 (0.15 − 1.11)	0.069
SMAD6	Surgery alone	380	1.17 (0.88 − 1.56)	0.28
	5-FU-based adjuvant	153	1.77 (1.23 − 2.55)	0.002*
	Other adjuvant	76	1.56 (0.65 − 3.78)	0.32
SMAD7	Surgery alone	380	1.64 (1.21 − 2.22)	0.0012*
	5-FU-based adjuvant	153	1.16 (0.82 − 1.63)	0.4
	Other adjuvant	76	1.8 (0.74 − 4.34)	0.19
SMAD9	Surgery alone	380	1.58 (1.17 − 2.14)	0.0029*
	5-FU-based adjuvant	34	1.67 (0.67 − 4.17)	0.27
	Other adjuvant	76	2.86 (1.14 − 7.19)	0.019*

*P < 0.05

Abbreviations: 5-FU, 5-Fluorouracil; CI, confidence interval; HR, hazard ratio.

**Table 5.** Correlation of SMAD gene expression level with overall survival (OS) of gastric cancer patients under different Her2 status

SMADs	Her 2 status	Cases	HR (95% CI)	P-value
SMAD1	Negative	429	0.48 (0.34 − 0.68	1.6e−05*
	Positive	202	0.66 (0.45 − 0.97)	0.035*
SMAD2	Negative	532	0.52 (0.4 − 0.66	1.5e−07*
	Positive	344	1.16 (0.89 − 1.5)	0.27
SMAD3	Negative	532	1.57 (1.22 − 2.03)	0.00039*
	Positive	344	1.73 (1.33 − 2.25)	3.5e−05*
SMAD4	Negative	532	0.64 (0.51 − 0.8)	0.00013*
	Positive	344	0.72 (0.55 − 0.93)	0.012*
SMAD5	Negative	532	1.37 (1.09 − 1.72)	0.0061*
	Positive	344	1.32 (0.99 − 1.75)	0.057
SMAD6	Negative	532	1.47 (1.17 − 1.84)	0.00071*
	Positive	344	1.44 (1.09 − 1.9)	0.01*
SMAD7	Negative	532	1.52 (1.2 − 1.92)	0.00044*
	Positive	344	0.8 (0.62 − 1.04)	0.089
SMAD9	Negative	429	1.85 (1.39 − 2.47)	2.1e−05*
	Positive	202	1.7 (1.16 − 2.51)	0.0064*

*P < 0.05

Abbreviations: HER2, Human Epidermal Growth Factor Receptor-2; CI, confidence interval; HR, hazard ratio.

The results indicated that SMAD1 expression was elevated in stage 1 and stage 3. SMAD2 and SMAD4 expression was elevated in stage 1 and stage 3. On the other hand, expression levels of SMAD3, SMAD5, SMAD6, and SMAD7 were downregulated in stage 2 and stage 3. Stage 4 is characterized by high progression of cancer, and therefore, only SMAD3, SMAD5, and SMAD9 showed prognostic significance in this stage.

High expression level of SMAD1 showed better prognostic value in gastric cancer with moderate differentiation. High expression level of SMAD3 was correlated with poor OS in moderate and well-differentiated gastric cancer. In addition, SMAD6 and SMAD7 were associated with poor OS in poorly differentiated gastric cancer.

The expression level of SMADs was not significantly correlated with OS of gastric cancer patients in different genders.

Different treatment strategies are associated with expression of SMADs. Surgery alone increased the expression levels of SMAD1, SMAD2, and SMAD4. On the contrary, treatment with 5-FU-based adjuvants decreased the expression levels of SMAD3 and SMAD6 but increased the expression of SMAD5. Furthermore, a high expression level of SMAD9 in patients treated with other adjuvants was associated with adverse effects.

The Her2 gene is associated with breast cancer. Expression levels of SMADs were not correlated with Her2 status.

### Inhibition of SMAD3 expression suppresses gastric cancer proliferation

**Figure 9.**
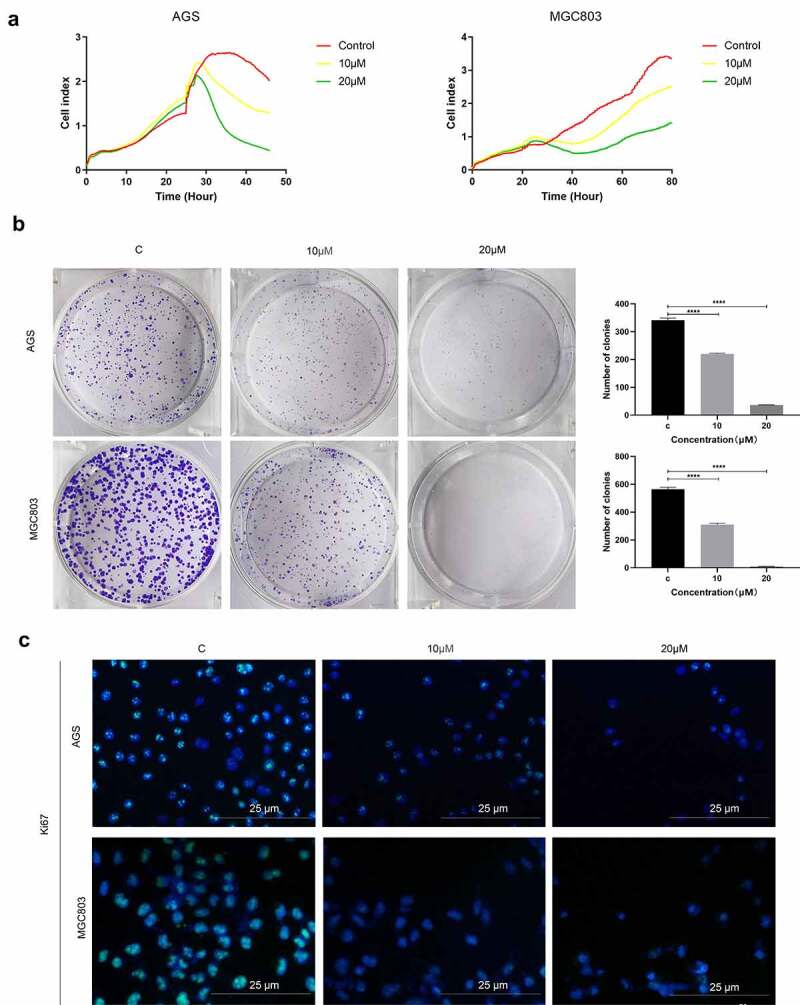
(e)-SIS3 inhibits proliferation of gastric cancer cells

To explore the role of SMAD3 in gastric cancer, gastric cell lines were administered with SMAD3 inhibitor (E)-SIS3. Label-free Real-time Cellular Analysis (RTCA) showed that cell proliferation ability of AGS and MGC803 (Figure 9(a)) was significantly inhibited by (E)-SIS3 concentration in a dose-dependent manner compared with DMSO solvent control group. Colony formation assay showed that (E)-SIS3 significantly inhibits proliferation and colony formation of AGS and MGC803 cells (Figure 9(b)). Ki67 Immunofluorescence analysis showed that proliferation of AGS and MGC803 cells decreased significantly after treatment with 10 μM and 20 μM (E)-SIS3 (Figure 9(c)). These findings imply that (E)-SIS3 treatment inhibits proliferation of gastric cancer cells in a dose-dependent manner.

(A) Label-free Real-time Cellular Analysis (RTCA) of AGS and MGC803 cells incubated with (E)-SIS3 (0, 10, 20 μM). (B) The proliferation of AGS and MGC803 cells with or without (E)-SIS3 treatment analyzed by colony formation assay. (C) Ki67 Immunofluorescence following AGS and MGC803 cells incubated with (E)-SIS3 (0,10, and 20 μM). Scale bar = 25 μm. Data are presented as mean ± SD, N = 3; ****P < 0.0001, compared with control.

### (E)-SIS3 inhibits migration of gastric cancer cells

**Figure 10.**
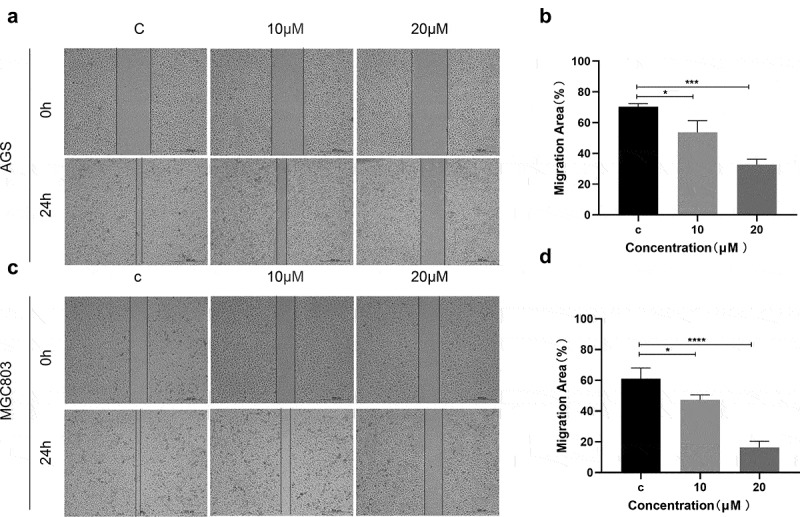
(e)-SIS3 inhibits the migration of gastric cancer cells

Furthermore, the ability of cell migration after (E)-SIS3 treatment was evaluated using wound healing experiments. Analysis of wound healing experiments showed that (E)-SIS3 significantly inhibited cell migration capacity of AGS (Figure 10(a,b)) and MGC803 cells (Figure 10(c,d)).

Migration of AGS (A and B) and MGC803 (C and D) cells incubated with 10 and 20 μM (E)-SIS3 or an equal volume of DMEM medium for 24 hours. (E)-SIS3 significantly inhibited migration of AGS and MGC803 gastric cancer cells. Scale bar = 500 μm. *P < 0.05; ***P < 0.001; ****P < 0.0001, compared with control.

### (E)-SIS3 induces gastric cancer cells apoptosis

**Figure 11.**
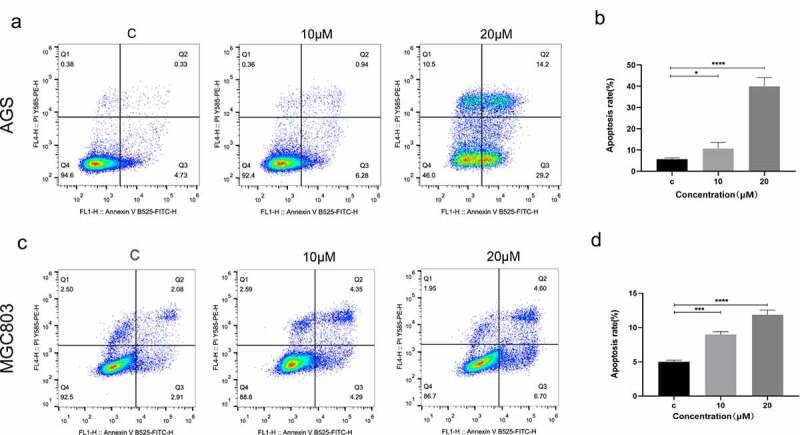
(e)-SIS3 induces apoptosis of gastric cancer cells

To explore the effects of (E)-SIS3 on apoptosis of gastric cancer cell lines, AGS and MGC803 cells were treated with 0, 10 and 20 μM (E)-SIS3 for 24 hours. Apoptosis was evaluated using annexin V/PI assay. Analysis showed that apoptosis rate of the 20 μM (E)-SIS3 treatment group was significantly higher compared with that of the control group. (E)-SIS3-induced apoptosis rate increased from 5.06% to 43.4% in AGS cells (Figure 11(a,b)) and from 4.58% to 11.3% in MGC803 cells (Figure 11(c,d)).

Flow cytometry results for apoptosis [apoptosis ratio was calculated as (Q2+ Q3)/(Q1+ Q2 + Q3+ Q4)] of AGS (A and B) and MGC803 (C and D) cells incubated with 10 μM and 20 μM (E)-SIS3 or an equal volume of DMEM medium for 24 hours. (E)-SIS3 significantly induced apoptosis in AGS and MGC803 cells. (E)-SIS3 induced apoptosis in a concentration-dependent manner. Data are presented as mean ± SD, N = 5; *P < 0.05; ***P < 0.001; ****P < 0.0001, compared with control.

### (E)-SIS3 inhibits the expression of SMAD3 genes in gastric cancer cells

**Figure 12.**
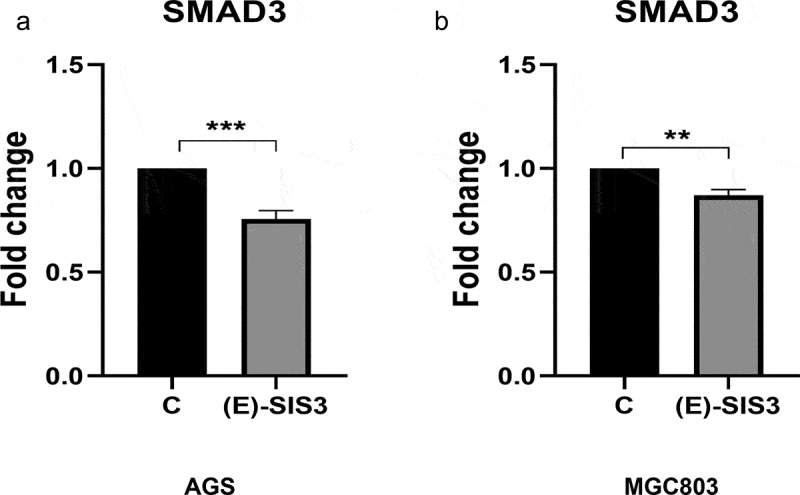
(e)-SIS3 inhibits proliferation and induces apoptosis of gastric cancer cells through influencing SMAD3 gene expression

In order to further verify the inhibitory effect of (E)-SIS3 on gastric cancer cell SMAD3, we measured the mRNA levels of SMAD3 gene in two gastric cancer cell lines treated with (E)-SIS3. RT-PCR results showed that the expression of SMAD3 mRNA in AGS (Figure 12(a)) and MGC803 (Figure 12(b)) cells decreased after being treated with (E)-SIS3 for 24 h. This confirmed that (E)-SIS3 is an effective inhibitor of SMAD3.

(A) The expression changes of SMAD3 genes in the AGS cell of gastric cancer after treating with (E)-SIS3 for 24 hours. (B) Changes in expression of SMAD3 genes in the MGC803 cell after treatment with (E)-SIS3 for 24 hours. Data are presented as mean ± SD, N = 3; **P < 0.01; ***P < 0.001; ****P < 0.0001, compared with control.

## Discussion

In this study, the prognostic significance of SMAD family in gastric cancer was explored using KM plotter. Analysis showed that high expression levels of SMAD1, SMAD2, and SMAD4 are associated with a better survival rate of gastric cancer patients, whereas SMAD3, SMAD5, SMAD6, SMAD7 and SMAD9 are associated with poor prognosis. Interestingly, expression levels of the SMAD family were not associated with mixed gastric cancer except the expression level of SMAD9.

SMAD1 is a substrate of mitogen-activated protein kinases and plays an important role in transferring signals from bone morphogenetic proteins. Therefore, it is implicated in various diseases like prostate cancer, fibrosis, and cardiovascular diseases. A study by Feng Qu reports that the growth, invasion, and migration of cancer cells can be inhibited through the ALK2/SMAD1 pathway [17]. Furthermore, a previous study reported that SMAD1 affects transcription regulation by forming a complex with SMAD4 [18]. This explains why high expression level of SMAD1 was associated with better OS. Transforming growth factor-beta (TGF-β)/SMAD signaling pathway plays important role in hepatic fibrosis. SMAD2/3 and SMAD4 represent stimulatory SMADs of this signaling pathway [19]. Activated SMAD2 and SMAD3 play different roles in cell growth, differentiation, and other biological functions [20]. SMAD2 mainly promotes growth and differentiation of hepatocytes, whereas SMAD3 promotes morphological and functional maturation of hepatic stellate cells [21,22]. In addition, SMAD3 is implicated in abdominal aortic aneurysm, osteoarthritis, mesenchymal cell transition, and cardiac fibrosis [23–26]. The role of SMAD3 in different cancer types has not been fully explored. A previous study reported that overexpression of SMAD3 decreases inhibition of melatonin in gastric cancer cells [27]. However, in the current study, SMAD3 was negatively correlated with survival rate. *In vitro* cell experiments were used to further explore the effects of SMAD3 on proliferation, apoptosis, and migration of AGS and MGC803 cancer cells. Analysis showed that (E)-SIS3 significantly inhibits proliferation and migration of AGS and MGC803 cells by down-regulating expression of SMAD3 and inducing AGS and MGC803 cell apoptosis. Although the mechanism should be further explored, these findings imply that smad3 is an important target for the treatment of gastric cancer. Analysis showed that expression of SMAD4 may lead to increased expression of p15 and induction of the activity of p15. Tob1 was silenced through specific siRNA. After a series of reactions, we observed a decrease in expression of cyclin D1, cyclin-dependent kinase-4 (CDK4), urokinase plasminogen activator receptor (uPAR), and peroxisome proliferator and activator receptor-δ (PPARδ) [28]. Low levels of these factors do not favor the growth of cancer cells, whereas high expression level of SMAD4 correlated with favorable OS. SMAD5 is a pH fluctuation messenger and plays a role as a regulator of physiological bioenergetic homeostasis by enhancing glycolysis by interacting with hexokinase 1 (HK1) [29]. In this study, high expression of SMAD7 was correlated with poor development of cancer. SMAD7 is a negative regulator of TGF-β signaling pathway, and it exerts anti-inflammatory effects by binding to Tables 2 and 3, thus inhibiting tumor necrosis factor (TNF) signaling pathway [30]. A study by Yi Yu reported that SMAD7 maintains cell pluripotency and modulates cytokine-dependent regulation of cancer and inflammation [31]. The high expression level of SMAD9 was associated with poor OS in all gastric cancer, gastrointestinal cancer, diffuse gastric cancer and mixed gastric cancer. This finding implies that SMAD9 may reflect the progression of gastric cancer. SMAD9 is overexpressed in different tissues and organs to regulate various cellular functions [32]. A previous study reports that SMAD9 is a transcriptional regulator in BMP signal transduction pathways [33]. Moreover, previous studies report that SMAD9 is implicated in gastrointestinal ganglioneuromas and follicular initiation [32,34]. These findings show that SMAD9 may be associated with the growth of cancer. Therefore, overexpression of SMAD9 is correlated with a poor survival rate in gastric cancer patients.

Japanese classification of gastric cancer is based on anatomy, especially the location of the lymph node [35]. In addition, the AJCC/UICC TNM classification is used to classify gastric cancer. This classification evaluates the depth of the primary tumor, lymph node involvement, and the probability of metastatic disease [36–38]. Staging is an important prognostic factor in various cancer types which can be used to guide treatment decisions. SMAD expression levels vary across different stages. SMAD1, SMAD2, and SMAD4 are associated with favorable OS in the first and third stage of gastric cancer. On the other hand, SMAD3, SMAD5, SMAD6, and SMAD7 were associated with poor OS in the second and third stage of gastric cancer. Gene expression level of SMADs showed inconsistent association with different differentiation stages. SMAD1 inhibits the growth, invasion, and migration of cancer cells. Therefore, SMAD1 is implicated in stage 1 to stage 3 of cancers. In addition, SMAD4 mainly affects cell cycle and is associated with better OS in stage 1 and cell differentiation.

Epidemiological studies report that regardless of etiologies, the incidence of gastric cancer in males is approximately two times higher compared with that of females [39]. The role of SMADs in males and females is the same, and therefore, gender does not affect the expression of SMADs. This is because SMAD function is not associated with the sex chromosome. However, the exact mechanism has not been fully elucidated.

Surgery is recommended for the treatment of most gastrointestinal cancer patients who should undergo curative resection. In addition, chemotherapy improves the curative resection (R0) rate and eliminates the micrometastatic disease [40]. 5-Fluorouracil (5-FU)-based chemotherapy, as a neoadjuvant, is an excellent choice for gastric cancer therapy. It functions in four ways. It incorporates fluorouridine triphosphate into RNA to interfere with RNA synthesis and function. In addition, 5-FU inhibits thymidylate synthase. Furthermore, 5-FU incorporates fluorodeoxyuridine triphosphate and deoxyuridine triphosphate into DNA. Furthermore, 5-FU activates programmed cell death signaling pathway through genotoxic stress. Moreover, 5-fluorinated pyrimidines have been widely used for the treatment of gastric, breast, pancreatic, colorectal cancers, and squamous cell cancers in the head and neck [41]. Therefore, we explored the effect of surgery, 5-FU-based chemotherapy and another adjuvant on expression levels of SMADs. Analysis showed that surgery showed favorable outcomes compared with other treatment approaches for patient’s high expression levels of SMAD1, SMAD2 and SMAD4. Patients with high expression of SMAD1, SMAD2 or SMAD4 showed better overall survival. Surgery showed minimal damage to healthy tissues compared with other treatment approaches. On the contrary, surgery alone may not be suitable for patients with high expression levels of SMAD7 and SMAD9. Administration of 5-FU-based adjuvants is recommended for patients with high expression of SMAD5. On the other hand, treatment with 5-FU-based adjuvants is not recommended for patients with high expression levels of SMAD3 and SMAD6. SMAD3 is mainly implicated in the morphological and functional maturation of cells. Mechanistically, 5-fluorinated pyrimidines may inhibit the functions of SMAD3.

Human epidermal growth factor receptor 2 (HER2) belongs to epidermal growth factor receptor (EGFR) family [42]. Mutations activate HER2 in HER2 or may result in receptor overexpression [32]. Previous retrospective studies report that HER2 is a positive prognostic factor associated with increased risk of invasion, metastasis, and poor survival. Therefore, HER2 can be used to diagnose all gastric cancer [26]. In this study, SMADs expression was not correlated with Her2 status. A previous study of 338 advanced cancer patients in the USA and Europe showed that, although the median overall survival in HER2-positive patients (13.9 versus 11.4 months, p = 0.047) was higher in the univariate analysis, the prognostic value was not observed in multivariate analysis [43]. The development of gastric cancer is affected by a myriad of factors, thus pinpointing that the role of one factor in prognosis of gastric cancer is challenging. This explains why HER2 showed no prognostic value in this study. Furthermore, the findings of this study show the potential role of (E)-SIS3 in gastric cancer therapy as it inhibits gastric cancer growth by regulating the SMAD system.

## Conclusion

This study shows that high expression level of SMAD1, SMAD2, and SMAD4 is associated with favorable OS of gastric cancer patients. In addition, we found that SMADs have limited prognostic value in the fourth stage of gastric cancer. Furthermore, SMAD3 is a negative prognostic factor of highly differentiated cancer, and association with male and female gender. In addition, surgery shows excellent therapeutic effect on patients with high expression of SMAD2, whereas 5-FU therapy is correlated with favorable OS for patients with high expression of SMAD5. Moreover, Her2 status was not correlated with SMADs expression level in gastric cancer. The findings of this study show that SMADs regulate the differentiation of cancer and provide information that will help to choose correct treatment strategies. Although the findings of this study show that SMAD expression is correlated with OS in gastric cancer patients, further studies should be performed to fully understand the mechanisms of SMADs.

## Abbreviations

SMAD, small mother against decapentaplegic; TGF-β, transforming growth factor-β; KM plotter, Kaplan-Meier plotter; RTCA, real-time cellular analysis; NCCN, National Comprehensive Cancer Network; OS, overall survival; HR, hazard ratio; DMSO, dimethyl sulfoxide; ATCC, American Type Culture Collection; BSA, bovine serum albumin; mRNA, messenger RNA; GC, gastric cancer; 5-FU, 5-Fluorouracil; HER2, human epidermal growth factor receptor-2.

## Highlights

1. The expression of SMADs is related to the OS of gastric cancer patients.

2. SMADs may play an important role in regulating cancer differentiation.

3. Inhibition of SMAD3 inhibited the proliferation of gastric cancer cells, migration and promoted apoptosis.
